# Everything is connected: Inference and attractors in delusions^☆^

**DOI:** 10.1016/j.schres.2021.07.032

**Published:** 2022-07

**Authors:** Rick A. Adams, Peter Vincent, David Benrimoh, Karl J. Friston, Thomas Parr

**Affiliations:** a Centre for Medical Image Computing, Dept of Computer Science, University College London, 90 High Holborn, London WC1V 6LJ, UK; b Max Planck Centre for Computational Psychiatry and Ageing Research, University College London, Russell Square House, 10-12 Russell Square, London WC1B 5EH, UK; c Sainsbury Wellcome Centre, University College London, 25 Howland St, London W1T 4JG, UK; d Department of Psychiatry, McGill University, H3G 1A4 QC, Canada; e Wellcome Centre for Human Neuroimaging, University College London, 12 Queen Square, London WC1N 3AR, UK

**Keywords:** Delusions, Active inference, Paranoia, Bayesian, Computational psychiatry, Choice-induced preference change

## Abstract

Delusions are, by popular definition, false beliefs that are held with certainty and resistant to contradictory evidence. They seem at odds with the notion that the brain at least approximates Bayesian inference. This is especially the case in schizophrenia, a disorder thought to relate to decreased – rather than increased – certainty in the brain's model of the world. We use an active inference Markov decision process model (a Bayes-optimal decision-making agent) to perform a simple task involving social and non-social inferences. We show that even moderate changes in some model parameters – decreasing confidence in sensory input and increasing confidence in states implied by its own (especially habitual) actions – can lead to delusions as defined above. Incorporating affect in the model increases delusions, specifically in the social domain. The model also reproduces some classic psychological effects, including choice-induced preference change, and an optimism bias in inferences about oneself. A key observation is that no change in a single parameter is both necessary and sufficient for delusions; rather, delusions arise due to conditional dependencies that create ‘basins of attraction’ which trap Bayesian beliefs. Simulating the effects of antidopaminergic antipsychotics – by reducing the model's confidence in its actions – demonstrates that the model can escape from these attractors, through this synthetic pharmacotherapy.

## 1 Introduction

Delusions are a puzzling phenomenon for all that encounter them, but for those who believe the brain performs inference according to Bayesian principles (or at least comes close to doing so), they must be especially mysterious. How could an inference engine that attempts to weight its prior expectations and sensory evidence optimally – according to their relative precision – generate beliefs that are not merely false, but held with undue certainty, and highly resistant to contradictory evidence (Williams, 2018)?

In this paper, we argue that the resolution to this puzzle lies in our brains' use of an internal model, not just of what is ‘out there’, but of how we act. Actions include movement, to change our immediate surroundings, but also mental or covert actions (Limanowski and Friston, 2018; Pezzulo, 2018); like the deployment of attention or selecting one hypothesis over another. This licences the notion of attractor states in habit formation – the tendency to repeat actions that have previously been selected – and to model delusions as resulting from the acquisition of a ‘mental habit’. This is motivated by the idea that inferences about the world may be conditioned on (i.e., depend on) the actions we select (Friston et al., 2017a; Stachenfeld et al., 2017), and so habits may give rise to inferences that are confidently held and insensitive to sensory evidence, much in the same way as habits themselves.

We illustrate this idea using numerical simulations and show how false inferences – and even delusions – can arise from moderate changes in the parameters of an internal or generative model. The ensuing active inference model also accounts for related phenomena, such as the influence of choices on subsequent inferences (Brehm, 1956), an ‘optimism bias’ in inferences about oneself (Sharot and Garrett, 2016), and, finally, a computational mechanism of action of antipsychotic drugs.

First, we must specify the aspects of delusions that we are trying to explain. This is because delusions are so heterogeneous that both supporting and contradictory evidence for almost any theory can be found among them. Definitions of delusions are problematic for the same reasons, but a popular definition includes, “a false belief based on incorrect inference about external reality that is firmly sustained despite what almost everyone else believes and despite… evidence to the contrary” (American Psychiatric Association, 2000). This study uses these ‘behavioural’ criteria – i.e., that delusions are i) beliefs (in the probabilistic sense) derived from inferences, ii) false (but see footnote 5), iii) held with great certainty, and iv) impervious to contradictory evidence – although ultimately, more mechanistic and formal criteria would be preferred.

In addition, we aim to reproduce not just these formal properties of delusions, but also their most common content, as this provides clues about their mechanisms. Most delusions have powerful affective themes: for example, of persecution, grandiosity, love, jealousy, guilt, or nihilism. Bleuler, who contributed much to the study of psychosis, including the term ‘schizophrenia’ itself, pointed out that “affects inhibit… contradictory associations and facilitate those that serve their purposes”. He held that delusions “develop under the dominance of one or several of the most important human drives”, citing love, power, wealth and fear of persecution as examples (Bleuler, 1950). Indeed, grandiose and persecutory delusions are associated with positive and negative affective states respectively (Knowles et al., 2011; Murphy et al., 2018), and there is evidence that anxiety and negative affect have a causal role in the formation of persecutory beliefs (Ben-Zeev et al., 2011; Brown et al., 2019). We therefore incorporate affect and mood in the model, and focus on delusions about the trustworthiness of others. In particular, we couple decisions about whether to trust or distrust others with calm or aroused affective states, respectively.

Recent computational work on delusions has focused on modelling the behaviour of subjects performing probabilistic inference tasks. This work is reviewed in detail elsewhere in this special issue (Ashinoff et al., 2021), but its implications can be summarised as follows. In general, sequential belief updating tasks have found that people with a diagnosis of schizophrenia (PSz) tend to make bigger belief updates than controls to *unexpected* information, which can also manifest as increased ‘switching’ (from one response to another) in bandit-type tasks (Fear and Healy, 1997; Garety et al., 1991; Langdon et al., 2010; Moritz and Woodward, 2005; Peters and Garety, 2006; Waltz, 2017; Young and Bentall, 1997). Computational models explain this effect in various but similar ways: as greater reversal probability (Schlagenhauf et al., 2013), belief instability (Adams et al., 2018), volatility (Cole et al., 2020; Deserno et al., 2020; Kreis et al., 2021; Reed et al., 2020), or non-linear (Stuke et al., 2017) or all-or-none updating styles (Nassar et al., 2021). Conversely, belief updating to *expected* or consistent information seems reduced in PSz (Adams et al., 2018; Averbeck et al., 2010; Baker et al., 2019; Nassar et al., 2021; Reed et al., 2020), meaning it can take longer for PSz to acquire new contingencies (Waltz and Gold, 2007).

These results can be interpreted under a simple model of how states of the world evolve to generate outcomes: namely, a hidden Markov model. Using such a model, an agent can infer the (hidden) states of the world, such as other people's intentions, from observed outcomes, such as receiving helpful or unhelpful advice (Behrens et al., 2008). The agent can also infer whether those states are changing over time. The probabilistic mappings between states and outcomes – *p*(*o*_*t*_| *s*_*t*_) – and between states over time – *p*(*s*_*t*+1_| *s*_*t*_) – are known as the likelihood and transition probabilities, respectively. In hierarchical models, the likelihoods can be regarded as mapping between hierarchical levels, where the outcomes of one level are the hidden states of the level below.^2^

One way of interpreting the above results is that the brain's model of the world is less precise, i.e. more uncertain, in PSz (Adams et al., 2013; Sterzer et al., 2018). Greater likelihood uncertainty means that small (expected) changes in inferred outcomes have less impact on belief updating about latent states, while greater transition uncertainty means that the persistence of states over time is less certain, hence unexpected outcomes make the agent *more* likely to infer that states of affairs have changed completely (e.g. that a contingency has switched). Belief updating tasks in the social domain have also found evidence of increased uncertainty about others' intentions in PSz, and in the (likelihood) mapping between those intentions and observed behaviour (Barnby et al., 2020a), although group differences in volatility are not always seen (Henco et al., 2020).

A critical point to note, however, is that although the above belief-updating abnormalities seem to be present in PSz, they have mixed relationships to delusions specifically (Ashinoff et al., 2021). Behaviourally, delusions sometimes relate to increased switching (Moritz and Woodward, 2005; Waltz, 2017) but not always (Langdon et al., 2010; Peters and Garety, 2006). Model parameters promoting updating in light of *unexpected* information sometimes correlate with delusions (Jardri et al., 2017; Stuke et al., 2017) or paranoia (Reed et al., 2020), as do those that resist updating, i.e. increased weight on priors (Baker et al., 2019). However, often no such relationships to delusion scores are found (Adams et al., 2018; Averbeck et al., 2010; Deserno et al., 2020; Nassar et al., 2021). Taken together, this work suggests that the brain's model of the world (in terms of ‘domain-general’ likelihood and transition probabilities) is more uncertain in PSz, and that this is maybe relevant to delusional beliefs, but it seems unlikely to be the only causal computational explanation.

This brings us to another somewhat baffling aspect of delusions. What is known of cortical pathology in schizophrenia – aberrant synaptic gain and disinhibition in neural networks (Krystal et al., 2017) – fits well with the idea that the brain's model of the world is imprecise. But how then can such a model develop beliefs that are so precise that they are incorrigible (Corlett et al., 2010)?

Below we show that various contributory factors – present to some degree in all individuals, healthy or otherwise – are in fact able to induce false inferences and, in some cases, delusional beliefs, as defined above. These factors include affect, the learning of priors over policies or ‘habitual’ learning and its effects on inference, and confidence in policies (policy precision). In the presence of reduced likelihood precision, various combinations of these interacting factors can push an agent into a delusional state. (For simplicity, we do not model effects of reduced transition precision here. However, this reduction would make inferred states more uncertain, and, in principle, lead to the same effects). From a technical perspective, the nature of (Bayesian) belief updating – under any generative model – necessarily induces conditional dependencies among all the model parameters. This means that changes in the estimate of any one parameter necessarily induces changes in other parameters to a greater or lesser extent. In turn, this leads to a kind of (computational) ‘diaschisis’ (i.e., focal pathology leading to network-wide disruption) that may play a special role in the formation of delusions.

We first describe the task the computational agent performs, and the active inference agent itself. We then show how changes in model parameters can lead to false inferences and delusions, and finally demonstrate a potential mechanism of action – in computational terms – for antidopaminergic treatments for delusions.

## 2 Methods

### 2.1 Experimental task

**Fig. 1:**
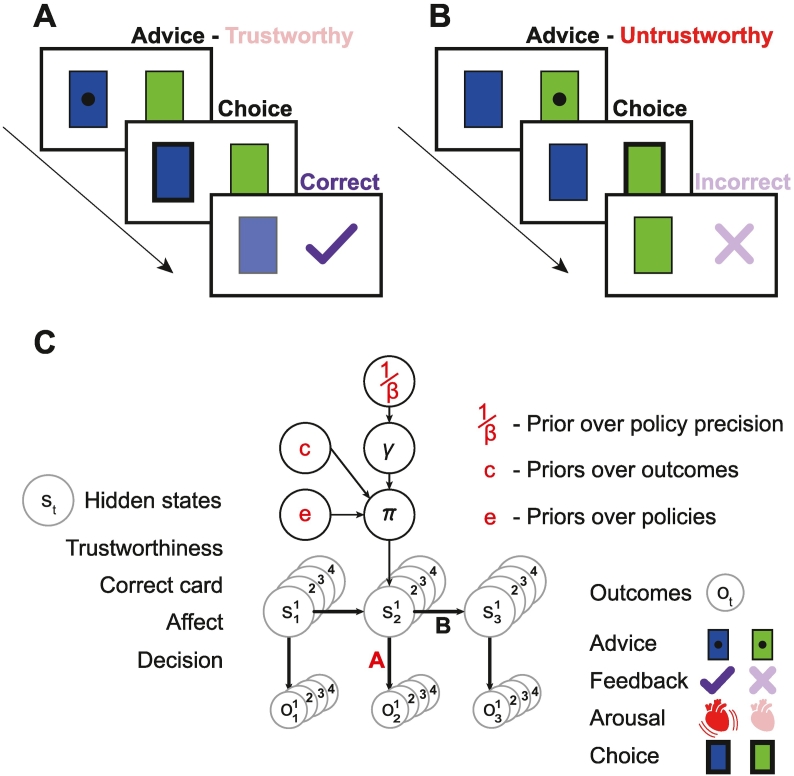
Task and model structure. A – The sequence of events within one trial, consisting of three timesteps. First, the agent receive advice to choose the blue card. Unbeknownst to the agent, the advisor is ‘trustworthy’. Next, the agent chooses the blue card, and then gets ‘correct’ feedback. B – The sequence of events with an ‘untrustworthy’ advisor. The agent follows the advice and gets ‘incorrect’ feedback. C – A schematic of the Markov decision process active inference model, during one trial (see the text and Supplement for a full description). Each trial consists of three timesteps. The exteroceptive and interoceptive outcomes *o* that the agent observes in each trial are shown at the bottom and listed on the right: they comprise the advice received, the feedback received, the arousal state (high or low), and the choice the agent makes. The agent must infer the hidden states *s* generating these outcomes: these states include the advisor's trustworthiness, the correct card, the agent's own affective state, and the agent's decision of which card to choose. The probabilistic (or deterministic) mappings from states to outcomes is given by the likelihood matrices in **A**. The transitions in hidden states across timesteps are given by the transition matrices in **B**. Some of these transitions depend on the agent's policy *π*, which is a sequence of control states *u* (e.g., ‘trust the advisor, choose blue’) across the three timesteps. The agent's choice of policy depends on its inferences about states *s* but also its priors over policies (or habits) *Dir*(**e**), its priors over outcomes (or preferences) **c**, and precision of (i.e., confidence in) its policies *γ*, which is heavily influenced by the prior over this precision, 1/*β*. For example, if an agent has trusted the advisor and/or chosen blue many times more than the other choices, its prior over these choices will be strengthened by the accumulation of counts in *Dir*(**e**). The agent is also strongly influenced by its priors over outcomes **c**, in which it expects to receive ‘correct’ feedback rather ‘incorrect’ feedback by a factor of exp(6). The agents with a negative ‘mood’ also predict ‘high’ arousal states to be more likely than ‘low’ (or vice versa, for positive mood). The precision over policies *γ* is continually updated, and denotes the agent's confidence that its policy will fulfil its priors over outcomes: a higher *γ* means it will choose its favoured policy more deterministically. The parameters coloured in red are later shown to contribute to false inferences: i.e., weaker likelihood (*a*) in **A**, and stronger influences of priors *Dir*(**e**), mood (*c*) in **c** and 1/*β*. Choice precision *α* is not shown. (For interpretation of the references to colour in this figure legend, the reader is referred to the web version of this article.)

The task (Fig. 1) is a simplified version of a paradigm used to probe both social and non-social inference (Behrens et al., 2008; Diaconescu et al., 2014). The agent tries to choose the correct card colour on each of 250 trials: blue or green. An advisor is present, who gives the agent advice about the correct card on each trial. The advisor can either be ‘trustworthy’ or ‘untrustworthy’: in which case the advice will be correct or incorrect with 90% probability, respectively. The sequence of events in each trial is: at timestep 1, the trial begins, at timestep 2, the agent receives some advice, and at timestep 3, the agent chooses a card and receives feedback (‘incorrect’ or ‘correct’).

A key simplification – in this version of the task – is that each trial begins anew with, in effect, a new advisor and new card decks. This means there is no sequential inference, and thus no need for a hierarchical model encoding the contingencies or accompanying volatility. This change was made in order to make the active inference model as simple as possible: hierarchical models – modelling state transitions across trials – should, in principle, give similar results (see Discussion). The correct card colours are random on each trial in all simulations. The advisor is trustworthy throughout all 250 trials in some simulations, and 50% trustworthy in the other simulations (starting in the untrustworthy state).

### 2.2 Active inference

Active inference is a Bayesian framework which treats both perception and action as inference problems (Botvinick and Toussaint, 2012; Friston et al., 2013). Perception is inference on the hidden states of the world causing sensory outcomes, and action is the result of inferring what policies (sequences of actions) must be adopted to obtain certain sensory outcomes. These inferences require a generative model *m* that encodes the probabilistic relationships between observed outcomes $\tilde{o}=\left[o_{1},\ldots ,o_{T}\right]$, hidden states of the world that the agent must infer $\tilde{s}$, policies *π*, and the sensory outcomes the agent expects, given its prior preferences $P\left(\tilde{o}|c\right)$. The model takes the form of a (partially observable) Markov decision process (MDP): a formalism used to model decision-making in agents that have a degree of control over some variables in their environment.

Beliefs about states are optimised by minimising free energy *F*, which can be expressed as follows (Friston et al., 2013):

(1) $$F=D_{\mathrm{KL}}\left[Q\left(\tilde{s},\pi \right)||P\left(\tilde{s},\pi |\tilde{o}\right)-ln\;P\left(\tilde{o}\right)\right]$$

The first term on the right-hand side means that *F* is minimised when *Q*, the agent's posterior belief about hidden states and policies, approximates the true distribution *P*. Optimising these beliefs about hidden states is the process of perceptual inference. Optimising beliefs about policies requires a slightly different approach because *F* is defined with respect to the past and present. In contrast, policy selection requires minimising free energy in the future *G*(*π*), i.e., the free energy expected under the predicted outcomes of a given policy *π*, denoted by posterior predictive beliefs *Q*. This can be expressed as:

(2) $$G\left(\pi \right)=-E_{Q}[D_{\mathrm{KL}}\left[Q\left(\tilde{s}|\tilde{o},\pi \right)||Q\left(\tilde{s}|\pi \right)\right]-E_{Q}\left[\ln P\left(\tilde{o}|c\right)\right]$$

The first term on the right-hand side describes the information gained by adopting a policy that reveals certain outcomes. The second term comprises the agent's preferred outcomes. Thus, the agent will choose policies that either reduce its uncertainty or achieve its goals. The ensuing prior belief over policies *P*(*π*) = *Cat*(***π***_0_) is determined by the expected free energy *G*(*π*) and a policy precision *γ*:

(3) $$\pi_{0}=\sigma \left(-\gamma ∙G\right)$$

Here *σ* is a softmax function, for which *γ* is the scaling parameter, and ***G*** is a vector whose *i*th element corresponds to *G*(*π* = *i*). Posterior beliefs about policies also incorporate *F*(*π*) – this means that policies minimising both immediate and expected free energy will be favoured:

(4) $$\pi =\sigma \left(-F-\gamma ∙G\right)$$

Unlike typical softmax parameters, *γ* is not fixed but optimised trial by trial (see Section 2.3.5). For a full description of the active inference framework used in this modelling please see Friston et al. (2017a).

The active inference model is also equipped with a choice precision parameter, *α*. This operates in exactly the same way as a standard softmax inverse temperature parameter, in that it controls the stochasticity of action selection. Unlike *γ*, it has no effect on inference, because it does not affect policies.

### 2.3 The generative process and model

The generative process describes how sensory outcomes are determined by the contingencies of the task (and agents' responses). The generative model is a model of this process, used by the agent to infer the hidden causes of its sensory outcomes and to select policies. The process and model contain both exteroceptive outcomes, i.e., the state transitions that constitute the task, and, crucially, interoceptive outcomes, that underwrite inferences about the affective state of the agent (Allen et al., 2019; Clark et al., 2018; Seth and Friston, 2016; Smith et al., 2019). We now describe the model in more detail (for the full model, please see the Supplement):

#### 2.3.1 Likelihood: mapping states to outcomes

Likelihood mappings of *p*(*o*_*t*_| *s*_*t*_) are encoded by **A** and **a** matrices, where upper case refers to the process generating data, and lower case to the brain's model of this process. **A**{1} maps deterministically from the hidden states of advisor intention (trustworthy or untrustworthy) and correct card (blue or green) to the outcome of advice received (blue or green). **A**{2} maps from the hidden states of correct card (blue or green) and choice made (blue or green) to some feedback outcomes (correct, incorrect or null (before feedback is received)) with 90% probability. **A**{3} maps deterministically from a hidden affective state (angry or calm) to an interoceptive outcome of arousal (high or low). This was absent in some simulations. Finally, **A**{4} maps deterministically from the hidden state of decision (null, blue or green) to a proprioceptive outcome of choice made (null, blue or green). All matrices are identical in the generative process (**A**) and in the generative model (**a**), except for **a**{2} – discussed in Section 2.3.4.

#### 2.3.2 Transitions: mapping states across timesteps

The transition matrices in **B** and **b** describe how the five hidden states evolve within a single trial, from timesteps one to three. Some are policy-dependent, i.e., change depending on the agent's choice of policy, defined as a sequence of control states *π* = {*u*_0_, …, *u*_*T*_}. Thus, a transition matrix for hidden state factor *n* is defined as **B**{*n*}(*i*, *j*, *u*) = *p*(*s*_*τ*+1_^*n*^ = *i*| *s*_*τ*_^*n*^ = *j*, *u* = *π*(*τ*)).

$B\left\{1\right\}=\left[\begin{array}{cc} 0.9 & 0.1\\ 0.1 & 0.9 \end{array}\right]$ corresponds to the advisor's intention, which changes from its initial setting (trustworthy or untrustworthy) at the start of the trial in 10% of trials. Hence in a sequence of 125 trials with a trustworthy advisor, incorrect advice will be dispensed on around 12–13 occasions. **B**{2} encodes the correct deck (blue or green), which does not change during the trial (it is an identity matrix). **B**{3} is a policy-dependent matrix that ensures the policy of deciding ‘blue’ or ‘green’ (or ‘null’, being undecided) maps deterministically to the decision states blue, green and null, respectively. In the models containing affect, **B**{4} is a policy-dependent matrix that makes a calm affective state (top row) twice as likely as an angry affective state (bottom row) if the agent decides to trust the advisor, and the converse if the agent decides to distrust the advisor:

(5) $$B\left\{4\right\}\;\left(u=\text{trust}\right)=\left[\begin{array}{cc} 2/3 & 2/3\\ 1/3 & 1/3 \end{array}\right]\;B\left\{4\right\}\;\left(u=\text{distrust}\right)=\left[\begin{array}{cc} 1/3 & 1/3\\ 2/3 & 2/3 \end{array}\right]$$

**B**{5} reflects the deterministic alternations in stages of the task, from null to advice to choosing/feedback stages. The transitions in the generative model **b** are the same, except for a policy-dependent version of **B**{1}, which means that if the agent chooses to trust the advisor, this makes them ‘trustworthy’ (top row), and conversely, if the agent chooses to distrust the advisor, this makes them ‘untrustworthy’:

(6) $$b\left\{1\right\}\;\left(u=\text{trust}\right)=\left[\begin{array}{cc} 1 & 1\\ 0 & 0 \end{array}\right]\;b\left\{1\right\}\;\left(u=\text{distrust}\right)=\left[\begin{array}{cc} 0 & 0\\ 1 & 1 \end{array}\right]$$

Trusting or distrusting the advisor is an inference about which (mental) policy was adopted and can be revised up until the final timestep.

To summarise, the agent's policies can affect transitions in two ways. First, it can choose a card. Second, it can choose to trust or distrust the advisor: in models containing affect, the former makes a calm state more likely, and the latter makes an angry state more likely. Note that the choice to trust or distrust the advisor is essentially a ‘mental’ action, akin to thinking “I am not going to believe anything you say, and I will be on my guard”.

We now turn to some aspects of the model that are crucial to the development of false inferences and delusions: affect, likelihood precision, policy precision, and habits.

#### 2.3.3 Priors over outcomes, including mood

The agent's expectations over the four sets of outcomes *P*(*o*_*T*_| **c**) are encoded in the **c** vectors. The agent has no preference about the advice outcomes **c**{1} (green, blue or null) or its observed choices **c**{4} (green, blue or null), so both are [0 0 0]. The agent prefers to receive ‘correct’ and not ‘incorrect’ feedback, and is neutral about ‘null’ feedback (prior to the choice), hence **c**{2} = [3 − 3 0]. The **c** vectors are log scaled, so the difference in preference between ‘correct’ (3) and ‘incorrect’ (−3) is a factor of exp(6). The **c**{3} vector [*c* − *c*] encodes moods, or the expected outcomes ‘low’ and ‘high’ arousal respectively (positive *c* values correspond to low arousal, i.e., positive mood). In models with neutral mood, the agent has no expectation over ‘low’ or ‘high’ arousal outcomes, so **c**{3} = [0 0]. In models with negative mood (*c* = − 1, for example), ‘high’ is more likely than ‘low’ arousal, so **c**{3} = [−1 1]. We term this ‘mood’ rather than ‘affect’, because it denotes a stable trait in the agent, as opposed to affective states which can vary across trials. Although we use the term ‘preference’ above, these are just descriptions of the distribution of outcomes our synthetic agent anticipates. Hence *c* = − 1 does not mean the agent likes having negative affect, rather that they expect to have negative affect more often than positive affect.

#### 2.3.4 Likelihood precision

If one's likelihood model of the world is imprecise, then the mapping between hidden states and sensory outcomes becomes less precise: i.e., likelihood precision is reduced (Benrimoh et al., 2018). We reproduce this effect in the agent's model by changing the precision of $a\left\{2\right\}=\left[\begin{array}{cc} a & 1-a\\ 1-a & a \end{array}\right]$, the mapping from hidden states of correct card and choice made to the feedback outcomes, from *a* = 0.6 (very imprecise) to *a* = 0.99 (very precise). As likelihood precision decreases, the feedback (‘correct’ or ‘incorrect’) has less effect on the agent's beliefs about which card was actually correct and whether the advisor lied.

#### 2.3.5 Policy precision

Policy precision *γ* determines the agent's confidence in selecting policies (Eq. (3)) and follows a gamma distribution parameterised by its prior 1/*β*_0_:

(7) $$\gamma =\frac{1}{\beta_{0}}$$

Its posterior estimate $\hat{\gamma }=1/\hat{\beta }$ (updated from trial to trial) is as follows (Friston et al., 2017a):

(8) $$\hat{\beta }=\beta +\left(\pi -\pi_{0}\right)∙G$$

This means that, if those policies whose probability goes up (from prior to posterior) correspond to those for which the expected free energy is most negative, the precision increases. More intuitively, if things are unfolding as anticipated, the precision or confidence placed in policy selection increases, and decreases otherwise. An alternative perspective is that policy precision reflects a reward prediction error (FitzGerald et al., 2015), where (negative) reward is expected free energy.

A crucial detail for what follows is that posterior beliefs about states are conditioned on policies (e.g., $Q\left(\tilde{s}|\pi \right)$ in Eq. (2)), which means that the most likely policy will make the biggest contribution to this posterior (through Bayesian model averaging):

(9) $$Q\left(s\right)=\sum_{\pi }Q\left(s|\pi \right)Q\left(\pi \right)$$

Because of this, policy precision can influence state inference, as its effect is to make particular policies (and the probable states under those policies) predominate. This is a key example of the conditional dependencies entailed by any form of belief updating. In this instance, beliefs about policies influence beliefs about states of affairs – and vice versa. This instance of conditional dependency is of particular interest because *γ* may be encoded by dopaminergic projections to the striatum (Adams et al., 2020; FitzGerald et al., 2015), and increased presynaptic availability of dopamine at striatal dopamine 2 receptors is a robust finding across schizophrenic and other psychoses (Howes and Kapur, 2009).

#### 2.3.6 Habits: learning priors over policies

The final aspect of the model – that is important for delusion formation – is the learning of priors over policies, or habits.^3^ The Bayesian learning of priors over policies is very simple (Chen et al., 2020): the agent has a Dirichlet distribution *Dir*(**e**) that acts as a conjugate prior for the parameters of prior beliefs over its policies, which accumulates one count for a policy each time it is chosen – or, when this is uncertain, accumulates an amount proportional to the posterior probability that each policy was chosen. High initial values across *Dir*(**e**) (termed ‘habit resistance’) mean that newly accumulated counts have little impact. Note that accumulating counts in this way means that habits can continue to gain strength every time they are performed, no matter whether they result in preferred outcomes (Friston et al., 2016). This means that once established, habitual policies are increasingly independent of outcomes and beliefs, but beliefs continue to be influenced by these policies (Eq. (9)).

#### 2.3.7 Model simulations

All simulations were performed using Matlab R2020a (Mathworks, Inc). The notational conventions used here are designed to mirror those used in Matlab simulations of active inference, to facilitate translation between the model described here and the code itself. The details of all simulations are given in Table 1. When the ‘changing trustworthiness’ sequence (from Fig. 3, Fig. 4) was used, the initial numbers of ‘untrustworthy’ trials (termed ‘initial consistency’) were varied to assess whether delusion formation depended on having a certain amount of consistently trustworthy or untrustworthy trials at the beginning. To do this a pseudorandom number of trials were removed from the end of the first 125 trials and moved to the end of the sequence. The active inference scheme is part of the SPM academic software: http://www.fil.ion.ucl.ac.uk/spm/. The code for the generative model used here, and for all figures in the paper, is available at: https://github.com/PeterVincent96/MDP_Delusions.

**Table 1:** The five sets of simulations performed, each of n = 972 agents. Parameter values were selected pseudorandomly from the ranges shown.

Model	Figures	Frequency of delusions with thresholds^a^ of 66% (60%, 70%)	Parameters, their ranges, and (below) Spearman correlations with delusion scores					
			Likelihood precision $\left[\begin{array}{cc} a & 1-a\\ 1-a & a \end{array}\right]$ *a* = 0.60 − 0.99	Habit resistance *Dir*(**e**) = 2 − 600	Initial consistency 1–125 trials	Mood [*c* − *c*] = −4.5 − 4.5	Choice precision *α* = 0.5 − 2.75	Policy precision $\frac{1}{\beta }=0.25-1.75$
No affect, consistently trustworthy sequence	2C, 5A	0% (0%, 0%)	*ρ* = −0.54	*ρ* = −0.40			*ρ* = 0.022	*ρ* = 0.32
With affect, consistently trustworthy sequence	4D, 5B, 6A	1.3% (1.7%, 1.3%)	*ρ* = −0.63	*ρ* = −0.20		*ρ* = −0.008	*ρ* = 0.017	*ρ* = 0.47
No affect, changing trustworthiness sequence	5C	1.0% (1.7%, 0.6%)	*ρ* = −0.44	*ρ* = −0.38	*ρ* = −0.034		*ρ* = 0.037	*ρ* = 0.34
With affect, changing trustworthiness sequence	4E, 4F, 5D, 6B, 7A	3.2% (4.6%, 2.0%)	*ρ* = −0.64	*ρ* = −0.20	*ρ* = −0.032	*ρ* = 0.039	*ρ* = 0.017	*ρ* = 0.48
With affect, changing trustworthiness sequence, with treatment	6C, 7B	0.5%	*ρ* = −0.63	*ρ* = −0.20	*ρ* = −0.036	*ρ* = 0.034	*ρ* = 0.020	*ρ* = 0.47

a Thresholds for falsity, certainty and incorrigibility criteria – see Section 2.3.7 – with results from alternative thresholds for all three criteria given in brackets.

A crucial point to note is that the agent may make many incorrect choices of green or blue cards – especially if it does not acquire informative prior beliefs about the likely trustworthiness of advice it will receive – but its posterior inferences about the advisor's trustworthiness follow the advice, choice *and feedback*, and so ought to be accurate. We defined false inferences as trials in which the agent inferred the incorrect trustworthiness state was >50% likely.

To assess delusion-like inferences, we devised a ‘delusion score’, based on the traditional criteria of falsity, certainty and incorrigibility. The delusion score (minimum 0, maximum 3) was the sum of the proportion of posterior inferences about advisor trustworthiness that were incorrect, the mean confidence (from 0 to 1) in these false inferences, and the proportion that were followed by another false inference (i.e., they were unlikely to be subsequently corrected by evidence or by the agent's own stochasticity of choices). Our criteria for delusions ‘proper’ used the following thresholds: i) falsity: >66% inferences being false in the ‘consistently trustworthy’ sequence, or >33% inferences being false in the ‘changing trustworthiness’ sequences (because a fixed delusion would only get a maximum of 50% of these inferences wrong); ii) certainty: >66% of false inferences were made with >80% confidence; iii) incorrigibility: >66% of false inferences were followed by another false inference on the next trial. Alternative thresholds of >60% and >70% were also used, in a sensitivity analysis. All relationships between parameter values and performance measures are given as Spearman's *ρ* correlations (Table 1).

To simulate antipsychotic treatment once delusions are forming, we allowed the model to proceed as normal in all agents, but once an agent had made 10 false inferences, its policy precision 1/*β* was reduced according to 1/*β*^∗^ = (1/*β* − min (1/*Β*)) × 0.5 + min (1/*Β*), where *Β* is the set of all *β* values used. This reduces high values of 1/*β* much more than low values, simulating an antagonist drug having greater effects in those with greater receptor activity, but not reducing activity to below the population minimum.

## 3 Results

### 3.1 Habits and precisions can improve performance

Initially, we showed that learning priors over policies (i.e., habits) and precisions over policies and choices can benefit an agent. We used a very simple scenario, in which the advisor is consistently trustworthy (except on 10% of the 250 trials), and the agent does not have affective states.

**Fig. 2:**
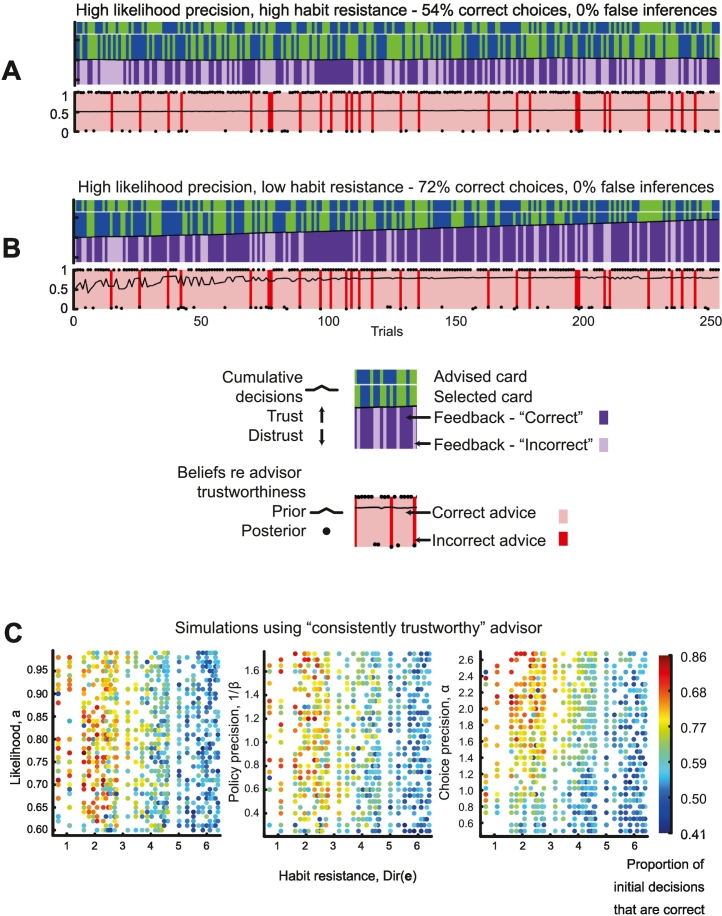
Habits and precisions improve performance. A – This plot shows the events and inferences in the task for all 250 trials, for an agent with high likelihood precision (*a* = 0.9), high habit resistance (*Dir*(**e**) = 600), a high prior over policy precision (1/*β* = 1) and moderate choice precision (*α* = 1.5). The advisor is ‘consistently trustworthy’, at *p* = 0.9. The band at the top shows the card advised by the advisor (blue or green). The second band down shows the card chosen by the agent (blue or green), and the third band shows the feedback received (dark purple is correct, light purple is incorrect). The black line between the second and third bands shows the cumulative decisions by the agent, of whether to trust (rising) or distrust (falling) the advisor: when it remains flat, as here, it means they are consistently trusting around 50% of the time. The colours in the bottom band show whether the advisor was trustworthy (pink) or not (red) on that trial. The agent's prior beliefs about the trustworthiness of the advisor on each trial are plotted as the black line in the bottom band. No habits are accumulated, so they remain at *p* = 0.5. The agent's posterior beliefs (following its choice and the feedback) are plotted as the black dots on the same axes: the agent tends to be certain that the advisor was trustworthy (*p* ≈ 1) or untrustworthy (*p* ≈ 0) on that trial. It does not make any false inferences. B – This plot shows the events and inferences in the task for an agent with low habit resistance (*Dir*(**e**) = 2), but other parameters identical to those in Fig. 2A. The advisor remains ‘consistently trustworthy’, at *p* = 0.9. It is able to learn a habit of trusting the advisor, and this favoured policy promotes a prior belief in the advisor's trustworthiness (bottom panel, black line). On occasions when the advisor is untrustworthy, however, this prior is easily overridden by the ‘incorrect’ feedback. C – These panels show the results of 972 simulations using the same sequence of advised cards and advisor trustworthiness as in the above figures, but varying likelihood precision, habit resistance, policy precision and choice precision parameters (and random seeds). Each panel shows the proportion of correct card choices, varying from 41% (below chance) to 86% (very good), as a function of habit resistance (*Dir*(**e**)) on the x axes, and on the y axes, likelihood precision *a* – left, prior over policy precision 1/*β* – centre, and choice precision *α* – right. (For interpretation of the references to colour in this figure legend, the reader is referred to the web version of this article.)

In the first simulation, the agent does not acquire habitual responses because its ‘habit resistance’ is very high (*Dir*(**e**) = 600). The likelihood precision is high (*a* = 0.9), i.e., the agent regards feedback as 90% reliable. Policy precision prior $\frac{1}{\beta }=1$ and choice precision *α* = 1.5. The accompanying timeseries is shown in Fig. 2A.

Several points can be taken from Fig. 2A. First, as there is no hierarchical inference across trials, the agent is essentially just guessing whether to trust the advisor and therefore which card to choose on every trial, so only 53.6% of its card choices are correct, and the agent's cumulative ‘trust’ or ‘distrust’ decisions (black line between second and third bands) remain unchanged. Second, because it cannot form habits, it does not accumulate any knowledge of the advisor's trustworthiness across trials, so its prior beliefs about the trustworthiness hidden state remain at *p* ≈ 0.5 throughout (black line, bottom band). Third, because its high likelihood precision means that it trusts the feedback it receives, its posterior beliefs (black dots, bottom band) about the advisor's trustworthiness are 100% accurate and close to certainty (1 or 0) on every trial.

The advantage of learning prior beliefs over policies (habits) in a stationary environment can be seen in Fig. 2B, which shows an identical simulation except that habit resistance was reduced to very low values (*Dir*(**e**) = 2). Because the agent's inference about whether to trust the advisor can be revised up until the final timestep (after feedback is received), it learns that it is likely to trust the advisor. Note that *it is not updating beliefs about the advisor* from trial to trial (unlike hierarchical inference models of this task, e.g., Diaconescu et al., 2014): it is merely updating a prior over its policy of trusting or distrusting, and, because inference about states is conditioned on policies (Eq. (9)), the most frequently chosen policies dominate this inference. Therefore, as it accumulates decisions to trust the advisor (ascending black line between the second and third bands), 72% of its card choices are correct, and its prior over advisor trustworthiness (black line, bottom band) also increases to plateau at *p* ≈ 0.8.

The panels in Fig. 2C show a summary of results of 972 simulations using the same sequence of advised cards and advisor trustworthiness, but varying likelihood precision, habit resistance, policy precision and choice precision parameters (and random seeds). Each panel shows the proportion of correct card choices, varying from 41% (below chance) to 86% (very good), as a function of different combinations of parameters. From the plots, and from correlations between the parameters and proportion of correct card choices, it is clear that agents perform better – in a stationary environment – when they can form habits (*Dir*(**e**) vs proportion correct, *ρ* = −0.69), choose less stochastically (*α* vs proportion correct, *ρ* = 0.42), and have greater policy precision (1/*β* vs proportion correct, *ρ* = 0.17). Increasing precision of sensory feedback is of some benefit (*a* vs proportion correct, *ρ* = 0.09) but even agents with very unreliable models of the world (e.g., *a* = 0.62, first plot, bottom left) can still perform very well as long as they can form habits to guide them.

### 3.2 Habits and low likelihood precision lead to false inference

**Fig. 3:**
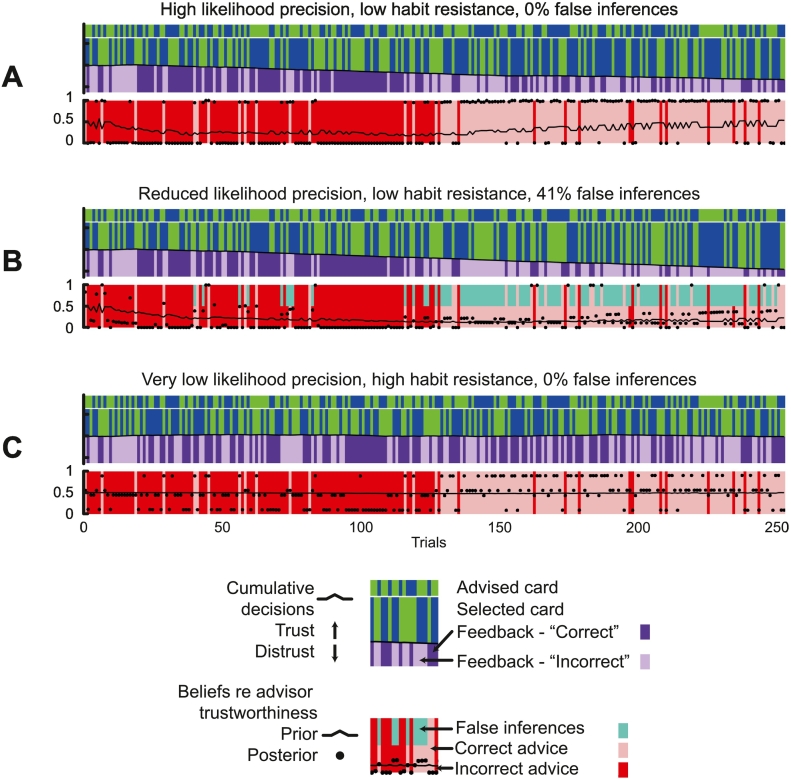
Habits and decreased likelihood precision can lead to false inferences. A – This plot shows the events and inferences in the task, in the same format used for Fig. 2, for an agent with high likelihood precision (*a* = 0.9), low habit resistance (*Dir*(**e**) = 2), a high prior over policy precision (1/*β* = 1) and moderate choice precision (*α* = 1.5), as in Fig. 2B. This time, the advisor has ‘changing trustworthiness’, with 125 trials at *p* = 0.1, and the next 125 trials at *p* = 0.9. The agent develops a habit of distrusting the advisor, and then trusting, and this change is reflected in its prior beliefs over trustworthiness (black line, bottom plot). B – This plot shows the events and inferences in the task for an agent with reduced likelihood precision (*a* = 0.75) but otherwise identical to the agent in Fig. 3A. Its reduced likelihood precision and learning of a habit of distrusting the advisor result in false inferences – defined as posterior beliefs about trustworthiness that are closer to falsity than truth, i.e., the wrong side of *p* = 0.5 – when the agent is actually being trustworthy. False inferences (defined as posteriors that the advisor is more likely than not to be trustworthy when in fact, they are untrustworthy, and vice versa) are indicated with cyan blocks on the bottom band. Although it makes many false inferences (41.2% of a possible 50%), they are not delusional by our criteria, because most are not within 0.1 of certainty (*p* = 0 or *p* = 1). C – This plot shows the events and inferences in the task for an agent with very low likelihood precision (*a* = 0.6) but also high habit resistance (*Dir*(**e**) = 600), otherwise identical to the other agents in Fig. 3. This agent does not learn any habit of trusting or distrusting, thus its prior over trustworthiness remains at *p* ≈ 0.5, and its posterior beliefs are always correct. The posteriors are either very uncertain (close to 0.5) or much more certain (far from 0.5): this depends upon whether the agent made the correct choice or not (see text). (For interpretation of the references to colour in this figure legend, the reader is referred to the web version of this article.)

The next set of simulations illustrates the performance of the habit-learning agent (*Dir*(**e**) = 2) in situations when the advisor changes from untrustworthy to trustworthy after 125 trials: parameter settings are as before, unless specified. In the first simulation (Fig. 3A), likelihood precision is high (*a* = 0.9), in the second simulation (Fig. 3B) it is reduced (*a* = 0.75), and in the third simulation (Fig. 3C) it is reduced even further (*a* = 0.6) but habit resistance is high (*Dir*(**e**) = 600).

The key point of the first simulation (Fig. 3A) is that – although by trial 125 the agent has developed sufficiently precise priors over its (distrusting) policies that it comes to believe the advisor is unlikely to be trustworthy (*p* ≈ 0.2, black line, bottom band) – when the advisor changes and becomes trustworthy, the agent's posterior beliefs over the advisor's trustworthiness are accurate (black dots, bottom band): it can override its prior because its likelihood is sufficiently precise. Indeed, its subsequent accumulation of trusting decisions then slowly shift this prior back to *p* ≈ 0.5 by the final trial.

In contrast, the agent in Fig. 3B shows dramatically different performance. The reduction in its likelihood precision means that once its habit of distrusting has lowered its prior over trustworthiness to *p* < 0.25 (around trial 40), the agent (optimally) trusts this prior over the sensory feedback, even when this feedback is ‘incorrect’. Thus, it begins to make false inferences about the advisor's trustworthiness (coloured cyan on the bottom band). This happens even before the contingency change on trial 125, after which they become far more frequent. The agent's posteriors do however drift back towards *p* = 0.5, and the false inferences stop by the final few trials.

The simulation in Fig. 3C shows that it is not low likelihood precision alone that causes false inferences, but its combination with learning priors over policies. In this simulation, likelihood precision is very low, but no false inferences occur, because the habit resistance is so high that *Dir*(**e**) does not affect the prior over trustworthiness, which remains at *p* ≈ 0.5 throughout.

A second interesting feature of these simulations is that the agent's posteriors over trustworthiness (black dots, bottom band) are sometimes very uncertain, i.e., close to *p* = 0.5, but sometimes much more certain, i.e., closer to 0 or 1. Looking at the purple band, one can see that the more certain inferences coincide with ‘correct’ feedback, and the uncertain inferences with ‘incorrect’ feedback. This is because without priors over policies or a precise likelihood to guide it, the strongest influence on the agent's posterior is the feedback from its own choice in that trial: the agent trusts ‘correct’ feedback much more than ‘incorrect’, *even when its choice was made arbitrarily*. Because the agent believes it will receive ‘correct’ feedback with far greater probability than ‘incorrect’ (in its prior beliefs over outcomes, Section 2.3.3), it becomes much more likely to trust the former, when other sources of information are unreliable.

### 3.3 Affect and mood

**Fig. 4:**
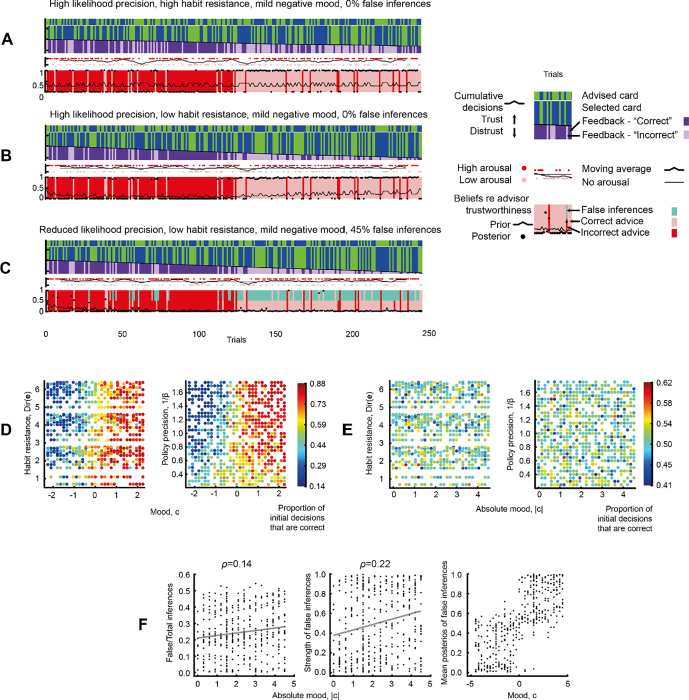
Mood's effect on inference and false inference. A – This plot shows the events and inferences in the task, in the same format used for Fig. 2, Fig. 3, for an agent with high likelihood precision (*a* = 0.9), high habit resistance (*Dir*(**e**) = 600), a high prior over policy precision (1/*β* = 1), moderate choice precision (*α* = 1.5), and with mildly negative mood (*c* = − 1). As in Fig. 3, the advisor has ‘changing trustworthiness’, with 125 trials at *p* = 0.1, and the next 125 trials at *p* = 0.9. The agent's arousal outcomes are plotted just above the bottom band as red dots (high arousal) and pink dots (low arousal), and the local mean over 10 trials is plotted as the black line. The agent's priors over trustworthiness do not change fundamentally because it cannot acquire habits of trusting or distrusting, but they do fluctuate according to its arousal, and are closer to ‘untrustworthy’ (i.e., 0) because of the agent's negative mood (see text). B – This plot shows the events and inferences in the task for an agent with low habit resistance (*Dir*(**e**) = 2) but otherwise identical to the agent in Fig. 4A. Given it can form habits over trusting behaviour, and given its negative mood, it develops a strong prior that the advisor is untrustworthy from trials 1–125 (compare with Fig. 3A, to which it is identical save for the addition of affect and negative mood). Nevertheless, it does not make false inferences because its likelihood precision remains high. C – This plot shows the events and inferences in the task for an agent with reduced likelihood precision (*a* = 0.75) but otherwise identical to the agent in Fig. 4B. The moderate reduction in likelihood precision has a drastic effect; it quickly develops such a strong prior belief that the advisor is untrustworthy that this overwhelms the (less precise) feedback and posterior inferences become likewise almost certain, even following 125 trials of largely trustworthy behaviour. The false inference trials are shown in cyan. This qualifies as a delusion according to our criteria, as it is false in >33% (of a possible 50%) trials, of >80% certainty in 66% of those false inferences, and >66% of false inferences were followed by another false inference. D – This panel shows the influence of mood and either habit resistance (left) or policy precision (right) on the proportion of initial decisions (i.e., whether to follow the advice) that are correct, in 972 simulations using different parameter settings. The sequence used was the ‘consistently trustworthy’ sequence from Fig. 2. It is clear that mood (with positive values indicating an expectation of low arousal, i.e., positive mood, and negative values high arousal or negative mood) has a very strong effect on these decisions – with positive mood boosting trusting behaviour, which is correct in this sequence – although habit resistance and policy precision also play a role. E – This panel shows results from 972 simulations similar to the previous panel, except that the ‘changing trustworthiness’ sequence was used, and absolute mood values are plotted on the x axes. Not surprisingly, the benefits of positive mood during the trustworthy period are cancelled out during the untrustworthy period, and vice versa for negative mood, so the net benefit of mood in this non-stationary sequence is zero. No parameter has a deleterious effect on choices overall, however. F – This panel shows how mood affects false inference in the simulations using the ‘changing trustworthiness’ sequence. The left-hand plot shows the weak correlation between absolute mood strength and the proportion of false inferences in the total. The middle plot shows the slightly stronger correlation between absolute mood strength and the strength (certainty) of false inferences, from 0 (maximally uncertain) to 1 (certain). The right-hand plot shows the very strong influence of mood (positive to negative) on the direction of false inference (trusting to distrusting). No correlation is given because the relationship essentially depends on the sign of the mood rather than its strength, as the previous plot shows. (For interpretation of the references to colour in this figure legend, the reader is referred to the web version of this article.)

The contribution of affect and mood to trustworthiness inferences – using the same sequence as Fig. 3 – is shown in Fig. 4A–C.

In Fig. 4A, the agent has a precise likelihood (*a* = 0.9) and does not form habits (*Dir*(**e**) = 600). It now has an affective state: its high (red dots) and low (pink dots) arousal outcomes are plotted just above the trustworthiness inferences, with the smoothed mean plotted between them. The agent has a slightly negative mood: *c* = − 1. Therefore, policies that lead to high arousal outcomes also become more probable: these policies simultaneously cause transitions in the advisor hidden state to ‘untrustworthy’, and the affective hidden state to become ‘angry’.^4^

In Fig. 4B, the agent is essentially the same as in Fig. 3A, i.e., with high likelihood precision (*a* = 0.9) and the ability to form habits (*Dir*(**e**) = 2), but in addition, negative mood (*c* = − 1). The habits compound the effect of the negative mood, making the prior on trustworthiness close to 0 by trial 125: more extreme than in Fig. 3A. Nevertheless, the high likelihood precision precludes false inference.

In Fig. 4C, likelihood precision drops such that *a* = 0.75 – as in Fig. 3B, but with negative mood as above. This has a drastic effect on posterior inferences: 45.2% (of a possible 50%) are false, because the combined effect of habits and negative affect push the prior on trustworthiness to such a low value that the model very rarely selects any action other than ‘distrust’, thus reinforcing the prior over policies still further, despite persistent evidence to the contrary. These inferences, which are i) false, ii) of great certainty, and iii) impervious to counterevidence, meet our criteria for a delusion.

Fig. 4D demonstrates that mood can have an enormous effect on correct card choices, i.e., the initial decision of whether to trust or distrust the advice. The plots are generated from 972 simulations of the model incorporating affective states, using the ‘consistently trustworthy’ sequence from Fig. 2, and varying other parameters. Unsurprisingly, positive mood encourages the agent to make (correct) trusting decisions, and the converse for negative mood. Mood's correlation to the proportion of correct choices is far stronger than the other parameters': mood *ρ* = 0.85, habit resistance *ρ* = −0.15, with likelihood precision *a*, *α*, and 1/*β* all having *ρ* < 0.1.

In the sequence in which trustworthiness changes halfway (used in Fig. 4A–C), however, having a constant mood is of little benefit. Fig. 4E shows the results of 972 simulations using this sequence: no parameters have more than weak relationships with the proportion of correct choices (all *ρ* < 0.1, including both mood and its absolute value). Fig. 4D and E show that mood can be of great benefit to inference, as long as the environment is sufficiently stationary (or, in theory, if mood changes to cohere with the environment).

Fig. 4F illustrates the effects of mood on false posterior inferences about the advisor, taken from the same simulations as Fig. 4E. The first plot shows that the absolute strength of mood (even up to very large values: surpassing the agent's preferences for being correct vs incorrect) has a relatively weak correlation with the number of false inferences made by each agent that made them (n = 407/972): *ρ* = 0.14. The second plot shows that absolute mood has a slightly stronger effect on the average strength (certainty) of these false inferences, *ρ* = 0.22. The third plot shows that mood has an enormous effect on the direction of false inferences: negative mood is almost always associated with distrusting false inferences, and the converse. Overall, it is clear that mood's main contribution to false inference is its ‘direction’ or theme, rather than its frequency or certainty. Affect, however, has much more pronounced effects on delusion-like inference: detailed in the next section.

### 3.4 Overall model and parameter relationships to delusion-like inferences

**Fig. 5:**
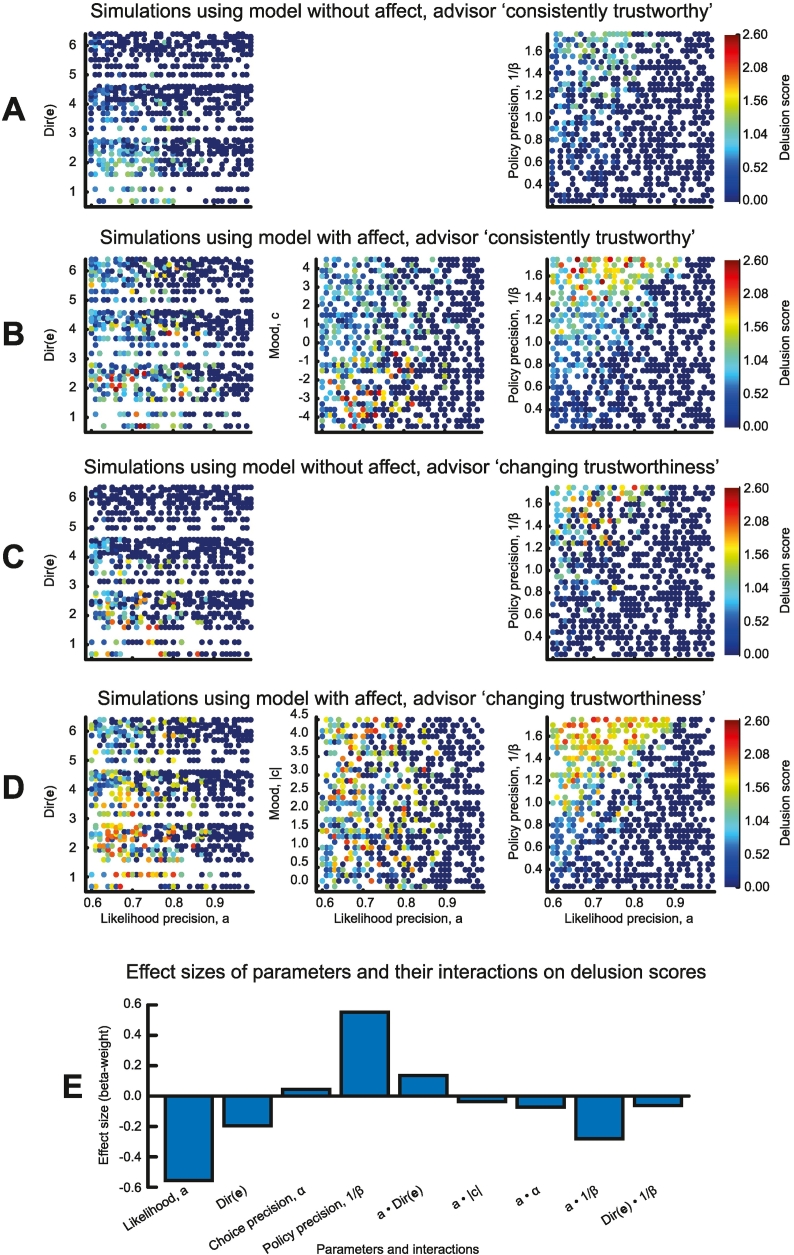
Parameter relationships to false inference in models with and without affect. A–D – These panels show the relationship of ‘delusion score’ (from 0 to 3, derived from the proportion of false inferences, and their certainty and incorrigibility: see Methods) to various parameters of different model simulations. Each dot is one simulation, with delusion score in colour, likelihood precision on the x axes and habit resistance, mood and policy precision on the y axes, in the same format, except for plotting mood in B and absolute mood in D. Only parameters with moderate-large effects on false inferences are shown. The models in A and C did not contain affective states, and those in B and D did. A and B used the ‘consistently trustworthy’ sequence employed in Fig. 2, C and D used variants of the ‘changing trustworthiness’ sequence used in Fig. 3, Fig. 4. Note that this means that if one extreme posterior over trustworthiness is consistently applied throughout, the maximum proportion of false inferences is 100% in the ‘consistently trustworthy’ sequence, and 50% in the ‘changing trustworthiness’ sequences. E – This bar plot illustrates the relative effect sizes (standardised regression beta weights) of the different parameters and interactions between parameters on delusion score in the model containing affect and evaluating the ‘changing trustworthiness’ sequence (Fig. 5D).

We now unpack the relationships among the model parameters and delusion-like inference in more detail (also see Table 1). Fig. 5 shows the ‘delusion scores’ (see Methods) for different parameter values (only parameters with moderate–large effects on delusion-like inferences are shown).

From the model without affective states and using the ‘consistently trustworthy’ sequence (Fig. 5A), it is clear that delusion-like inferences are most likely at low likelihood precision, low habit resistance and greater policy precision – the latter reinforces the effects of habits (priors over policies) on inferences – but no delusions ‘proper’ occur.

Introducing affect into this model dramatically increases the proportion of delusion-like inferences for some parameter settings (Fig. 5B), and causes delusions ‘proper’ in 1.3%. In particular, negative mood encourages the model to infer ‘untrustworthiness’, which is incorrect in 90% of trials (middle panel). Nevertheless, it is also clear from the top of the middle panel that false inferences also arise under positive mood and low likelihood precision: many of these agents would meet criteria for delusions if the evidence were to change.^5^

In the ‘changing trustworthiness’ sequences (Fig. 5C and D), delusion-like inferences can occur at both extremes of trustworthiness. The relationships to model parameters – similar in all simulations – are most clearly seen in Fig. 5D: the strongest determinant of delusion score is likelihood precision (*ρ* = −0.64), then policy precision (*ρ* = 0.48), and habit resistance (*ρ* = −0.20). Absolute mood strength and choice precision have minimal effects on delusion score (*ρ* < 0.05), although the presence of affect in the model clearly encourages delusion-like inference (comparing Fig. 5C and D), and trebles the proportion of delusions (from 1.0% to 3.2%). The relative proportions of delusions remain consistent if alternative thresholds of 60% and 70% are used (see Section 2.3.7 and Table 1). In summary, the capacity for affective states substantially increases the frequency of delusions, whereas priors over the content of those affective states (i.e., mood) drive their content (Fig. 4F).

Fig. 5E shows the relative contributions of parameters and also interactions between parameters (all standardised for comparability) in a regression model predicting delusion scores, in the simulations from Fig. 5D. Only statistically significant betas are shown: note that absolute mood is not a predictor, but it interacts with likelihood precision – as do the other model parameters – and habit resistance interacts with policy precision.

Note that the delusion-like inferences simulated here are not simply ‘reversal learning’ deficits: i) many (up to 82% in one simulation) false inferences occur in Fig. 5B, where there is no reversal at all, and ii) there is almost no correlation between the initial consistency (i.e. number of ‘untrustworthy’ trials) in Fig. 5C and D and delusion scores (both *ρ* = −0.03) – one would expect these correlations to be higher if reversal learning deficits were the issue.

### 3.5 Delusions and their treatment

We now examine what proportion of agents with delusion-like inferences meet our criteria for delusions (see Section 2.3.7) – and how a treatment for delusions might work.

**Fig. 6:**
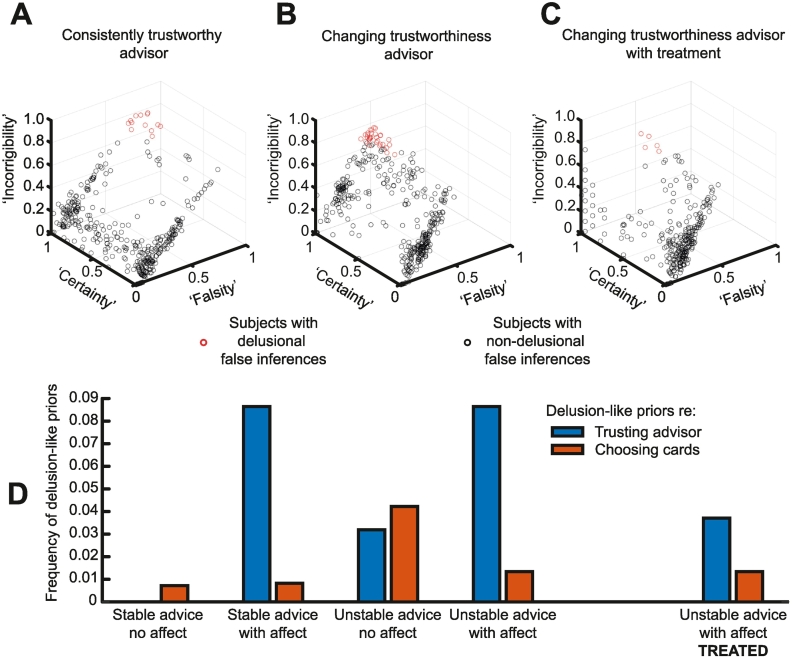
Delusions and their treatment. The top panels plot the characteristics of delusion-like inferences in only those agents that had them in various simulations. In each case, only a small proportion (in red) meet all three criteria for delusions, namely i) falsity: >66% inferences being false in the ‘consistently trustworthy’ sequence, or >33% inferences being false in the ‘changing trustworthiness’ sequences; ii) certainty: >66% of false inferences were made with >80% confidence; iii) incorrigibility: >66% of false inferences were followed by another false inference on the next trial (see Section 2.3.7). A – This plot shows the results of the 433/972 agents who developed false inferences in the model incorporating affect and using the ‘consistently trustworthy’ sequence (the same simulations shown in Fig. 5B). 13 meet criteria for delusions. B – This plot shows the results of the 407/972 agents who developed false inferences in the model incorporating affect and using the ‘changing trustworthiness’ sequence (the same simulations shown in Fig. 5D). 31 meet criteria for delusions. C – This plot shows the results of the 407/972 agents who developed false inferences in the same setup as Fig. 6B, but who were ‘treated’ by reducing their policy precision after 10 false inferences (see Section 2.3.7). 5 meet criteria for delusions. D – This plot shows the proportions of agents who develop delusion-like priors over policies (i.e., habits) concerning either choosing cards or trusting the advisor, in all five sets of simulations. Delusion-like priors about the card occur with roughly similar frequency to those about the advisor, except in models that incorporate affect, in which delusion-like priors about the advisor dominate. (For interpretation of the references to colour in this figure legend, the reader is referred to the web version of this article.)

Fig. 6A and B plot the characteristics of false inferences in the 433/972 and 407/972 agents that had them in the ‘consistently trustworthy’ and ‘changing trustworthiness’ sequences (respectively). In each case, only a small proportion meet all three criteria for delusions (as Fig. 4C would, but Fig. 3B would not): 13 in the former, and 31 in the latter.

One aspect of the model – we have not yet explored – is the possibility of it becoming deluded not about the advisor but about the correct card: either is possible if likelihood precision is reduced, because this makes feedback less informative about both the advisor and the card. The first four sets of bars in Fig. 6D illustrate that delusion-like priors over policies (defined according to the final distribution in *Dir*(**e**) as a >75% probability of choosing one card or one trust state over another, or of >50% probability of distrusting in the ‘consistently trustworthy’ sequence) about the card also occur, with roughly similar frequency to those about the advisor, except in models that incorporate affect. In the latter, delusion-like habits are much more commonly about the advisor than the card, because affective states are coupled to inferences about trustworthiness (via the linking of trusting and affective policies) in the model: as may be the case in many psychotic delusions.

Our final question was whether we could simulate the potential action of antidopaminergic antipsychotics. To do so, we reduced policy precision *γ* – thought to be encoded by striatal dopamine – after any agent made 10 false inferences, to simulate the initiation of treatment of delusion formation (Section 2.3.7). This reduction had a marked effect, reducing the number of delusional agents from 31 to 5 (Fig. 6C, also Fig. 6D). Note that this intervention does not resolve delusions just by making behaviour more stochastic (otherwise, one would expect negative correlations between choice precision *α* and false inferences, which are not seen in Table 1).

**Fig. 7:**
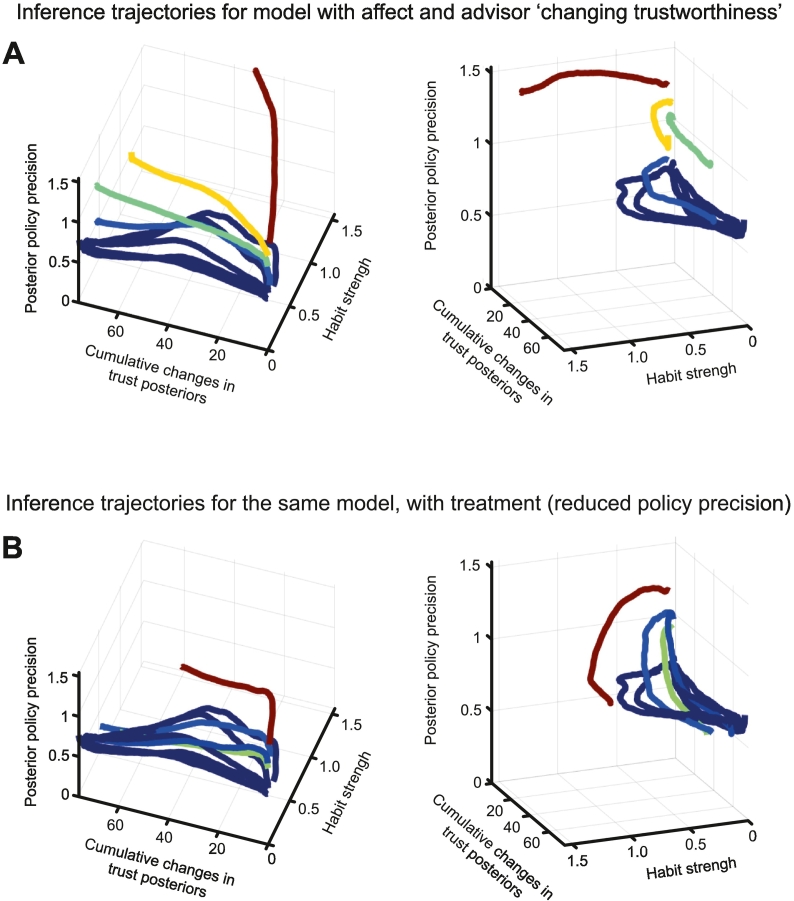
The evolution of inferences in the model state-space, and the effect of treatment. These panels show state-space plots of posterior policy precision *γ*_*Τ*_, habit strength (defined as ∣ ln (**e**(trust)_*T*_/**e**(distrust)_*T*_)∣) and cumulative trial-to-trial changes in posterior beliefs about trustworthiness at the final timestep *T* from trials 1–250. Agents were sorted in order of their proportions of false inferences, and deciles averaged together and plotted. As in Fig. 5, the colours indicate the average proportion of false inferences in each decile. The timeseries starts in the bottom right corner of the plots on the left. The plots on the right show the same data, but are rotated for viewing purposes. A – This plot shows the trajectories for the model with affect and the ‘changing trustworthiness’ sequence (as in Figs. 5D and 6B). B – This plot shows the trajectories for the same model, but with a ‘treatment’ of lowered policy precision applied after 10 false inferences (as in Fig. 6C). The drop in policy precision (more visible on the right plot) is then followed by substantially more cumulative belief updating (left plot), even though habit strength does not decline.

To assess how key variables in the model evolve over time in agents with different proportions of false inferences, and how they are affected by ‘treatment’, we created state-space plots of *γ*_*t*_, habit strength (defined as ∣ ln (**e**(trust)_*T*_/**e**(distrust)_*T*_)∣) and cumulative trial-to-trial changes in posterior beliefs about trustworthiness. Agents were sorted in order of their proportions of false inferences, and deciles averaged for plotting. Fig. 7A shows the trajectories for the model with affect and the ‘changing trustworthiness’ sequence. They fall into three groups: the blue trajectories, which develop habits to varying degrees but can then revise these habits – in part because their policy precision is lower – and continue to update their beliefs. The yellow and green trajectories make habits of roughly similar strength to the blue group, but are unable to revise them fully when the evidence changes, and are prone to false inference as a result. Last, the red trajectory quickly forms strong habits, even increasing its policy precision from its (high) initial value, and adjusts its beliefs very little.

The importance of policy precision in determining how beliefs evolve is evident both from the way it distinguishes the groups in Fig. 7A, and from the effect of decreasing it in Fig. 7B. Instead of increasing from its starting value, policy precision is forced downwards instead of increasing further (right plot), whereupon changes in posteriors from trial to trial rapidly increase (left plot), *even though habit strength does not decline*.

## 4 Discussion

In this paper, we have shown that apparently highly ‘non-Bayesian’ decision-making – such as the certainty and incorrigibility of delusions – can result from moderate changes in certain parameters of a Bayes-optimal agent. The key parts of the model that contribute to these false inferences are: reduced likelihood precision, which reduces the impact of sensory evidence; affect, which biases beliefs towards trusting or distrusting, and a propensity to form overconfident priors over policies (i.e., habits), itself determined both by low habit resistance and high policy precision. These parameters make both independent and synergistic contributions to false inference: in particular, reduced likelihood precision is necessary but not sufficient, and interacts with both mood and habit-forming tendencies.

Thus, in contrast to symptoms like compulsions, which have been ascribed to a single computational variable (Fradkin et al., 2020), delusions may be more akin to a ‘failure mode’: a specific dysfunction within a system that can have multiple causes, including the design of the system itself (Walters and Redish, 2018). This failure mode^6^ exists due to potential feedback loops or ‘attractors’ in the system, that cause self-maintaining states. One example here is that habits affect beliefs (because state inference depends on the most likely policies: Eq. (9)), and beliefs affect habits (chosen policies depend on inferred states). Likewise, beliefs drive affect, which drives beliefs (Hesp et al., 2021). Third, the development of habits (i.e., priors over policies) can increase the precision over policies (*γ*), and increased *γ* will drive consistent selection of that habit. This phenomenon is seen in Fig. 7A, in which a high initial value of *γ* grows even higher as habit strength also increases: this cycle is broken by reducing *γ* with ‘antipsychotics’, whereupon habit strength stops increasing and posterior beliefs undergo more updating (Fig. 7B). Note that the reduction of *γ* does not directly change policies or reduce false inferences: these occur because of the recurrent belief updating among *γ*, policies, habits and beliefs.

This delicate interplay is a necessary aspect of Bayesian belief updating; especially using schemes such as belief propagation and the variational – and neuronally computable – message passing employed in the simulations above. This follows because these are examples of Bayesian inference that entail conditional dependencies among all the unknown states and parameters of the model. Technically, when belief updating is framed as self-evidencing (Hohwy, 2016) – namely, optimising model evidence (or minimising variational free energy) – conditional dependencies are inevitable and shape the basins of attraction in the accompanying free energy landscape. One could conceive of these basins of attraction in terms of the above attractors – or indeed the parasitic attractors proposed in parallel distributed processing models of schizophrenia (Hoffman and McGlashan, 2001).^7^ In this work, we have explored the kind of priors that underwrite inference and choice behaviour characteristic of delusions. As with much previous work in this area, the parameters that cause psychopathology pertain to the precision of various (Bayesian) beliefs, under hierarchical generative models: see references reviewed in Friston et al. (2017a) and Friston et al. (2017b).

Importantly, habits and mood can improve performance (Fig. 2) – e.g. habits enable more consistent choices in stochastic environments (Schwöbel et al., 2021) or if one's model of the world is imprecise (Fradkin et al., 2020) – just as all prior beliefs can aid ill-posed inference problems. Difficulties arise, however, when likelihood precision is reduced to the extent that priors can dominate, and – further – push the system into dysfunctional attractor regimes. Note that in reality, many more potential attractors exist than are modelled here: e.g. beliefs changing mood (rather than just affect), which changes beliefs, behaviour of the agent affecting behaviour of the advisor and vice versa, which can even induce a folie à deux (Friston et al., 2020), and so on. Likewise, participants performing this task (Diaconescu et al., 2014) clearly use sequential and hierarchical inference (i.e., they infer change in a single advisor over time, and volatility in that change), which our model omitted for simplicity. However, one would expect all the parameters modelled here to play the same role in a hierarchical model: namely, reduced likelihood and transition precision –– and increased policy precision – would again bias inference away from sensory evidence and towards expectations based on habitual responding.

The idea of minds (and environments) as such multistable systems goes back to cybernetics (Ashby, 1952) and early psychological accounts of psychosis: e.g. of anxiety and threat generalization reinforcing each other (Mednick, 1958), and today is standard in cognitive accounts of paranoia (e.g. Bentall et al., 2001; Freeman, 2016) and also of negative symptoms (Strauss, 2021). It is challenging to model such processes within short experimental paradigms, but they may be key to explaining how relatively small differences in model parameters may develop into marked differences in behaviour (Robinaugh et al., 2019). These marked behavioural differences may appear to constitute distinct ‘disorders’ – e.g., see the isolated group of deluded agents in Fig. 6A – when in fact there is continuous variation in the underlying parameters.

We now comment on some specific aspects of the model. One important observation is that when likelihood precision is reduced, the prior belief that one will make correct decisions has a strong effect on evaluation of the feedback from that decision, even if the decision was arbitrary (Fig. 3C). This may explain the well-known phenomenon of ‘choice-induced preference change’, whereby choices subsequently increase one's valuation of that choice (Brehm, 1956). This occurs even following random choices, but not in choices allocated by computer (Sharot et al., 2010), and increases with confidence in choice and uncertainty over values (Lee and Daunizeau, 2020). ‘Cognitive dissonance’ theory was originally proposed as an explanation (Festinger, 1957), but a simpler account is that subjects are inferring from their own behaviour, given their priors (Bem, 1967) – indeed, post-judgement biases in perception can be modelled in a similar way (Luu and Stocker, 2018). This constitutes a fourth dysfunctional ‘attractor’ in this system: how choices affect inferences, and inferences affect future choices.

A second example of the intimate links between action and perception in active inference is the influence of priors over policies on inference over states (see Section 2.3.6). We have termed these priors ‘habits’, but note that here the habit learned is purely mental, without effects on the advisor. It essentially implies that consistently adopting an attitude of suspicion towards others will strengthen one's inferences that they are untrustworthy (Corlett et al., 2010). Auditory verbal hallucinations have been modelled similarly: adopting a conversational attitude to perceived voices may intensify them (Benrimoh et al., 2019, Benrimoh et al., 2018). Outside the active inference framework, there is neural and modelling evidence that state inference is sometimes conditioned on (especially habitual) policies: the best example is the ‘successor representation’ (Dayan, 1993), a prediction about forthcoming states (given the current state and policy) thought to be encoded by hippocampal place cell fields (Stachenfeld et al., 2017), which show clear policy-dependence, e.g. conforming to barriers necessitating detours (Alvernhe et al., 2011), habitual directions of travel (Mehta et al., 2000) or sampling of rewards (Hollup et al., 2001).

Thus, the precision over policies may be a crucial parameter in psychosis, because in reinforcing policies it also bolsters the likely states under those policies: delusions and hallucinations. This precision is thought to be encoded by striatal dopamine (FitzGerald et al., 2015) – supported by both fMRI (Schwartenbeck et al., 2015) and PET (Adams et al., 2020) imaging of humans performing tasks modelled using active inference – and dopamine 2 receptors (D_2_Rs) in the indirect pathway in particular, given they seem to reduce choice stochasticity by inhibiting competing actions (Cui et al., 2013; Eisenegger et al., 2014; Humphries et al., 2012; Kwak et al., 2014; Lee et al., 2015). Indeed, a recent landmark study (Schmack et al., 2021) has shown that in mice trained to nose poke and then wait for a reward if a tone is played (disguised by white noise), dopamine levels in the tail of the striatum covary with sensory expectations: i.e., the state conditioned on the mouse's policy. Strikingly, optogenetic stimulation of these dopamine neurons induces more ‘false alarms’ in the task, an effect abolished by the D_2_R antagonist Haloperidol.

A third case of action-perception interaction arises concerning actions in the interoceptive domain, i.e., affective responses, and their influence on beliefs. As with choice biasing subsequent inference, being in an affective state also biases inference about other states such as advisor trustworthiness, because they are coupled at the policy level. This is reminiscent of the well-known ‘optimism bias’ in beliefs about oneself (e.g. that you are less likely to get cancer than the average person), which is most pronounced when i) beliefs are motivated, i.e. tied to affective states, and ii) the likelihood is less precise (Sharot and Garrett, 2016). These mirror the conditions under which our simulated agent makes delusion-like inferences. The importance of the current affective state is underlined by the fact that perceived threat removes the optimism bias (Garrett et al., 2018), and merely increasing arousal in healthy people reduces perceived trustworthiness of faces (Abbott et al., 2018). The latter effect is exaggerated in PSz with persecutory ideas (Hooker et al., 2011).

Another likely contribution of affective state to delusions – not modelled here – is its facilitatory effect on habitual learning (Pool et al., 2021). Chronic stress causes medial prefrontal volume loss and makes decision-making less goal-directed and more habitual, in both rats (Dias-Ferreira et al., 2009) and humans (Soares et al., 2012). Stress may promote habits by making knowledge (here, likelihood precision) more uncertain (Schwabe and Wolf, 2009): indeed, working memory capacity protects against this effect (Otto et al., 2013). One can therefore clearly see how ripe the conditions are for habit (delusion) formation in early psychosis: greater uncertainty, altered affect, mounting stress, and a background of cognitive impairment. Furthermore, viewing delusions as forms of habitual learning suggests why, when psychotic episodes recur, old delusions typically return instead of new ones forming: habits likewise return on re-exposure to their previous contexts (Schwöbel et al., 2021; Wood and Neal, 2007).

Of all parameters in the model, reduced likelihood precision plays the most important permissive role in generating false inferences, both by itself, and through interactions with other parameters. This may seem at odds with previous work (Adams et al., 2013; Fletcher and Frith, 2009) showing that many phenomena in PSz (such as resistance to visual illusions, smooth pursuit eye movement deficits, reduced oddball EEG responses, etc.) can be explained by a loss of precision of *prior beliefs* relative to sensory precision (which ought to be attenuated, but is not). However, the likelihood *p*(*o*| *s*) in the current model encompasses the entire predictive coding hierarchy described previously,^8^ e.g., mapping hidden states to observations, etc., so uncertain prior beliefs in the former model equate to decreased likelihood precision in this decision-making agent.

In our model, the loss of likelihood precision was a ‘domain-general’ deficit. The reduced reliability of feedback applied equally to inferences about cards as well as trustworthiness, as both are informed by accurate feedback (Section 2.3.4). However, it created ‘domain-specific’ (i.e., social) delusions when affect was included in the model (Fig. 6D). This is because affect – here, coupled to decisions about trustworthiness – is a potent driver of the attractor states that are enabled by a generalised loss of likelihood precision. Notably, paranoia is associated with (domain-general) perceptual abnormalities, whereas social anxiety is not (Freeman et al., 2008), and paranoid individuals show belief updating patterns consistent with reduced likelihood (and transition) precision in neutral tasks (Reed et al., 2020), despite delusions themselves having typically strong affective themes. If moods (priors over affects) are more responsible for the themes rather than the general presence of delusional ideas (Fig. 4F), it makes sense that paranoia specifically – and not anxiety or interpersonal sensitivity – is associated with attributions of harmful intent (Barnby et al., 2020b). Indeed, persecutory delusions are associated with negative affect and low self-esteem (Murphy et al., 2018), rather than being psychological mechanisms for preserving positive self-esteem, as an early computational model of paranoia proposed (Colby, 1975). Likewise, grandiose delusions also tend to be consistent with prevailing affect, rather than defences against negative affect, as proposed by other theories (Knowles et al., 2011). Likelihood precisions may also play a role in certain delusional themes, of course: social inferences carry the most uncertainty (FeldmanHall and Shenhav, 2019).

One should question how biologically realistic the parameter ranges used here might be: a global decrease in likelihood precision from *a* = 0.9 to *a* = 0.6 is extreme and probably only found in dementias. Nevertheless, a less severe decrease to *a* = 0.75 still leads to delusions, and seems realistic, given many delusions also concern inherently uncertain domains. Conversely, very high habit resistance is also unrealistic: no one is immune to habits, so the population is likely at the lower end of the simulated range. The extremes of mood we have modelled, which slightly dominate other priorities, seem reasonable. It is hard to know what realistic policy precisions might be: we centred our range around the default value.

The criteria for delusions we have used here emphasise their continuity with paranoid persecutory beliefs in other disorders and the general population (Freeman, 2007). Indeed, at the computational level, paranoia in population samples resembles paranoia in PSz (Reed et al., 2020). The eminent psychopathologist Karl Jaspers felt our criteria were “mere external characteristics”, however, and that a better approach was to classify ‘delusion-like ideas’ as being understandable in terms of preceding affects, trauma or hallucinations, and ‘delusions proper’ as being “ununderstandable”, in that no psychological mechanism could account for either their origin or their subsequent incorrigibility (Walker, 1991). Here we have demonstrated a psychological mechanism that might explain incorrigibility, but otherwise, our agent's delusions are ‘understandable’. We have not touched on the “direct, unmediated [by thought], intrusive knowledge of meaning” that “entails a change in the totality of understandable connections” – i.e. a fundamental alteration in the structure of semantic knowledge – that Jaspers felt was the true pathology of psychosis (Jaspers, 1913). Such experiences are less common than ‘understandable’ delusions, however, even in schizophrenia (only 5–19% of admissions: Mellor, 1991).

Related to this, Bayesian model reduction could be used to adjudicate between competing explanations for a small number of observations, creating ‘ah-ha’ moments of (abductive) insight that are unmediated by conscious thought (Friston et al., 2017b). Although very data-efficient, this process can also overfit the data, leading to ‘superstitious’ inferences: this depends on the hypothesis space, and coincidences between the hypotheses and the data. Similarly, greater precision within a Dirichlet process mixture model can generate novel, over-fitted explanations for observations (Erdmann and Mathys, 2021). A complete account of delusions must assess whether these factors are sufficient to explain referential ideas (whose affective component is not always so clear), why delusions are not ‘corrected’ by memory and/or reasoning systems (related to the second factor in the ‘two factor’ theory of delusions: Coltheart and Davies, 2021), and especially why they seem immune to intersubjective norms – what Kant (1798/2012) termed the ‘sensis communis’ – which usually provide powerful constraints on beliefs (Bell et al., 2021).

In terms of future implications of this work, the Bayesian basins of attraction illustrated here may be best explored using detailed longitudinal (rather than cross-sectional) data, which calls for challenging modifications to experimental design: this issue applies across computational psychiatry (Huys et al., 2021). The model predicts that conditional dependencies of inferences about states – on affects, policies, and policy precision – ought to correlate with delusion scores. It also predicts that D_2_R antagonists reduce delusions by reducing policy precision rather than by reducing ‘aberrant salience’ (Kapur, 2003). Although these concepts are related (Adams et al., 2016): the former predicts antipsychotics ought to have relatively more impact on evidence relating to the dominant policy, rather than non-dominant policies (predicted by the latter). The model has interesting implications for psychological therapies too: it implies that purely behavioural treatments for delusions may work not just by extinguishing avoidance (e.g., learning that bad things won't happen if I meet others), but also by encouraging decisions and establishing new habits that themselves alter inferences about states (e.g., going to the café every day means it is safe).

## 5 Conclusions

Here, we have shown that delusional certainty and incorrigibility can arise in a Bayes-optimal active inference agent, through permissive changes in likelihood precision (making sensory feedback less reliable), policy precision and habit resistance (increasing its confidence in its actions and the states of the world implied by those actions), and affect (biasing inferences that are associated with affective states). Interactions between these parameters can cause the system to become trapped in dysfunctional attractor belief states – i.e., delusions – from which it is difficult to escape without help. One such mechanism of escape is the lowering of policy precision – the possible mechanism of action of antipsychotic drugs. The effects of decisions and effects on inferences in the model may also explain well-known psychological findings: choice-induced preference change and the optimism bias. Thus rather than being incompatible with Bayes, delusions may exist because of the self-reinforcing dependencies caused by Bayesian updating.

## Role of the funding source

The funders had no role in this work.

## Declaration of competing interest

DB is a shareholder and employee of Aifred Health, a digital mental health company. Aifred's work does not relate to the contents of the present article. The other authors have no competing interests to declare.

## Appendix A Supplementary data

Supplementary material Image 1

## Footnotes

2 In this paper we consider both the transition probabilities and likelihood mappings as empirical priors in a hierarchical generative model. This contrasts with the distinction between prior and sensory precision in treatments of Bayesian belief updating that focus on sensory likelihoods (Adams et al., 2013; Karvelis et al., 2018).

3 The habits acquired in active inference are slightly different from habits in reinforcement learning, e.g. the ‘model-free’ state-action pairing that is learned when the action is rewarded but persists when the action is devalued (Daw et al., 2005). In active inference, agents do not act to obtain rewards, but to fulfil their prior beliefs; habitual policies are therefore policies that the agent has learned it is most likely to pursue, irrespective of its belief state, rather than any value (Friston et al., 2016). In this and similar frameworks (Maisto et al., 2019; Schwöbel et al., 2021), priors over policies are combined with likelihoods within the planning-as-inference scheme (Attias, 2003; Botvinick and Toussaint, 2012).

4 An interesting detail is that the probabilistic nature of this latter transition (Eq. (5)) means that it is 33% probable that the agent adopts the ‘distrust’ policy and still finds itself in a ‘low’, not ‘high’, arousal outcome. This means that sensing a low arousal outcome is relatively weak evidence – weighed against the prior over affective state, *c* – against having pursued a policy of treating the advisor as untrustworthy: hence even in *low* arousal, this bad-tempered agent’s prior belief over advisor trustworthiness is slightly *less* than *p* = 0.5.

5 This illustrates the shortcomings of ‘falsity’ as a criterion for delusions (Spitzer, 1990), despite its usefulness in revealing their presence: given delusions are independent of sensory evidence, they cannot be defined with respect to that evidence.

6 Operationally, a ‘failure’ here means that there are other belief structures and priors that would provide a more apt account of exchange with the environment – and have a greater marginal likelihood (or smaller free energy).

7 Strictly speaking, these basins of attraction are Bayes optimal solutions to inference, even if they manifest as suboptimal. This follows from the complete class theorem (Brown, 1981) that states for any given pair of decisions and loss functions there exists some priors that render the decisions Bayes optimal.

8 To explain this point further, the predictive coding hierarchy used to model smooth pursuit and other paradigms in Adams et al. (2013) mapped from sensations to the hidden states generating those sensations (e.g., the oscillation frequency of a sinusoidal target). Thus, hidden states were at the top of the predictive coding hierarchy. In the present model, the observations represent a high-level summary of the inferences made by the predictive coding hierarchy that deals directly with sensory input. This means observations, from the perspective of our model, equate to estimates of hidden states from the predictive coding perspective. Therefore, imprecise representations of hidden states in predictive coding are best described as imprecise priors, but in the MDP—where those same hidden states are now considered observations, they result from imprecise likelihoods *p*(*o*| *s*) or transitions *p*(*s*_*t*+1_| *s*_*t*_).
