# Supplementary material for: Everything is connected: Inference and attractors in delusions
Source: Schizophr Res. 2022 Jul;245:5–22. doi: 10.1016/j.schres.2021.07.032 (PMC9241990; doi:10.1016/j.schres.2021.07.032)
Supplement: Supplementary file 1 — Supplementary material [file mmc1.docx]

Supplementary information

Everything is connected: inference and attractors in delusions

Rick A Adams^1,2^*†, Peter Vincent^3^*, David Benrimoh^4^, Karl J Friston^5^, Thomas Parr^5^

^1^Centre for Medical Image Computing, Dept of Computer Science, University College London, 90 High Holborn, London, WC1V 6LJ, UK

^2^Max Planck Centre for Computational Psychiatry and Ageing Research, University College London, Russell Square House, 10-12 Russell Square, London, WC1B 5EH, UK

^3^Sainsbury Wellcome Centre, University College London, 25 Howland St, London, W1T 4JG, UK

^4^Department of Psychiatry, McGill University, H3G 1A4, QC., Canada

^5^Wellcome Centre for Human Neuroimaging, University College London, 12 Queen Square, London, WC1N 3BG, UK

The structure of the MDP model is given below.

Hidden states and transitions: Transition matrices for the generative process are in capitals, the generative model is in lower case (only shown if different to the generative process).

$s^{1}$ – Hidden state of ‘advisor’ is trustworthy or untrustworthy:

$\mathbf{B}\left\{ 1,u=\mathrm{trust} \right\}=\left[ \begin{matrix} 0.9 & 0.1 \\ 0.1 & 0.9 \end{matrix} \right]$, $\mathbf{B}\left\{ 1,u=\mathrm{distrust} \right\}=\left[ \begin{matrix} 0.9 & 0.1 \\ 0.1 & 0.9 \end{matrix} \right]$

$\mathbf{b}\left\{ 1,u=\mathrm{trust} \right\}= \left[ \begin{matrix} 1 & 1 \\ 0 & 0 \end{matrix} \right]$, $\mathbf{b}\left\{ 1,u=\mathrm{distrust} \right\}= \left[ \begin{matrix} 0 & 0 \\ 1 & 1 \end{matrix} \right]$

$s^{2}$ – Hidden state of ‘correct card’ is blue or green:

$$\mathbf{B}\left\{ 2 \right\}=\left[ \begin{matrix} 1 & 0 \\ 0 & 1 \end{matrix} \right]$$

$s^{3}$ – Hidden state of ‘affect’ is calm or angry:

$\mathbf{B}\left\{ 4,u=\mathrm{trust} \right\}= \left[ \begin{matrix} 2/3 & 2/3 \\ 1/3 & 1/3 \end{matrix} \right]$, $\mathbf{B}\left\{ 4,u=\mathrm{distrust} \right\}= \left[ \begin{matrix} 1/3 & 1/3 \\ 2/3 & 2/3 \end{matrix} \right]$

$s^{4}$ – Hidden state of ‘decision’ is blue/green/null:

$\mathbf{B}\left\{ 3,u=\mathrm{blue} \right\}=\left[ \begin{matrix} 1 & 1 & 1 \\ 0 & 0 & 0 \\ 0 & 0 & 0 \end{matrix} \right]$, $\mathbf{B}\left\{ 3,u=\mathrm{green} \right\}=\left[ \begin{matrix} 0 & 0 & 0 \\ 1 & 1 & 1 \\ 0 & 0 & 0 \end{matrix} \right]$,$\mathbf{B}\left\{ 3,u=\mathrm{null} \right\}=\left[ \begin{matrix} 0 & 0 & 0 \\ 0 & 0 & 0 \\ 1 & 1 & 1 \end{matrix} \right]$

$s^{5}$ – Hidden state of ‘stage’ cycles through null, advice or decision:

$$\mathbf{B}\left\{ 5 \right\}=\left[ \begin{matrix} 0 & 0 & 1 \\ 1 & 0 & 0 \\ 0 & 1 & 0 \end{matrix} \right]$$

The likelihood mappings from hidden states to sensory outcomes:

$o^{1}$ – Outcome of ‘advice’ is blue/green/null, depending deterministically on $s^{1}$ and $s^{2}$ (and stage $s^{5})$:

When $s^{5}(2)=1$, if $s^{1}(1)=1$ then in $\mathbf{A}\left\{ 1 \right\}$, $o^{1}=s^{2}$, else $o_{1}=\neg s^{2}$

$o^{2}$ – Outcome of ‘feedback’ is correct/incorrect/null, depending on $s^{2}$ and $s^{3}$ (and stage $s^{5})$:

When $s^{5}(3)=1$, mapping from columns signifying $s^{2}=s^{3}$ and $s^{2}\neq s^{3}$ respectively, then (omitting null outcome): $\mathbf{A}\left\{ 2 \right\}=\left[ \begin{matrix} 0.9 & 0.1 \\ 0.1 & 0.9 \end{matrix} \right]$

In the agent, the precision of this matrix varies but always takes the form:

$\mathbf{a}\left\{ 2 \right\}=\left[ \begin{matrix} a & 1-a \\ 1-a & a \end{matrix} \right]$ where $0.6\leq a\leq0.99$

$o^{3}$ – Outcome of ‘arousal’ (interoception) is low or high, depending deterministically on $s^{3}$

In $\mathbf{A}\left\{ 3 \right\}$, $o^{3}=s^{3}$

$o^{4}$ – Outcome of ‘observed choice’ (exteroception) blue/green/null, depending deterministically on $s_{4}$

In $\mathbf{A}\left\{ 4 \right\}$, $o^{4}=s^{4}$

The prior preferences over outcomes are as follows:

$\mathbf{c}\left\{ 1 \right\}=[0 0 0]$ for $o^{1}$, advice outcomes blue/green/null

$\mathbf{c}\left\{ 2 \right\}=[3-3 0]$ for $o^{2}$, feedback outcomes correct/incorrect/null

$\mathbf{c}\left\{ 3 \right\}=\left[ c-c \right]$ where $-4.5\leq c\leq4.5$ for $o^{3}$, arousal outcomes low or high (i.e. $c$ encodes ‘mood’, or prediction of ‘low’ arousal over ‘high’ arousal)

$\mathbf{c}\left\{ 4 \right\}=[0 0 0]$ for $o^{4}$, decision outcomes blue/green/null

The policies $\pi$ are sequences of control states $u_{1:3}$ (from timesteps 1-3) that select the relevant $\mathbf{B}$ matrices, i.e. determine subsequent transitions. Four policies are possible: trust advisor, choose blue; trust advisor choose green; distrust advisor, choose blue; distrust advisor, choose green.
